# Choledocholithiasis: A Review of Management and Outcomes in a Regional Setting

**DOI:** 10.7759/cureus.50223

**Published:** 2023-12-09

**Authors:** Jason Zouki, David Sidhom, Rebecka Bindon, Tejminder Sidhu, Erick Chan, Matthew Lyon

**Affiliations:** 1 General Surgery, Toowoomba Hospital, Toowoomba, AUS; 2 General Surgery, Townsville Hospital, Townsville, AUS; 3 General Surgery, Gold Coast Hospital and Health Service, Gold Coast, AUS

**Keywords:** gallstones, common bile duct exploration, common bile duct stone, endoscopic retrograde cholangio-pancreatography, choledocholithiasis

## Abstract

Background

Choledocholithiasis is a common surgical presentation with an incidence of 8% to 16% in symptomatic cholelithiasis. Treatment often requires a multi-stage approach via endoscopic retrograde cholangiopancreatography (ERCP) and laparoscopic cholecystectomy (LC), which can prolong the length of stay (LoS) and expose patients to unnecessary risks. A single-stage procedure, such as LC with common bile duct exploration (CBDE), is a safe and effective option that may decrease LoS. This study compares patient outcomes and management in a regional center and aims to identify factors that predict the presence of confirmed choledocholithiasis.

Methods

A retrospective cross-sectional analysis was performed on all patients admitted to Toowoomba Hospital for management of diagnosed or suspected choledocholithiasis from January 2021 to March 2023. Patient demographics, ERCP findings, and operative data were collated.

Results

A total of 195 patients were identified, including 136 patients undergoing multi-stage management, 34 patients who had an ERCP alone, and 25 patients who underwent single-stage management. Single-stage procedures had an 80% success rate with an average LoS of 3.6 days. Multi-stage procedures had an average LoS of 8.1 days and an ERCP success rate of 93%. Complication rates between ERCP (11.7%) and LC with CBDE (9.7%) were comparable. Time to index ERCP and serum bilirubin level were found to be significantly lower in those with positive index ERCP findings compared to those without.

Conclusion

Single-stage procedures are a safe way to manage choledocholithiasis and are associated with a reduced LoS when compared to multi-stage management, with comparable efficacy and morbidity rates.

## Introduction

Choledocholithiasis is a common sequelae of gallstones, with an incidence ranging from 8% to 16% in patients with symptomatic gallstones [1]. Untreated choledocholithiasis can be a common cause of repeat presentations and can progress to life-threatening complications, including acute cholangitis or gallstone pancreatitis. Initial investigations for suspected choledocholithiasis typically follow a presentation of clinical jaundice or symptomatic cholelithiasis and include a comprehensive clinical examination, liver function tests (LFTs), and imaging. However, predicting choledocholithiasis is often difficult, and the diagnosis frequently presents as an incidental finding [2] on an intraoperative cholangiogram (IOC). The 2019 European Society of Gastrointestinal Endoscopy (ESGE) guidelines [3] outline several approaches to the management of choledocholithiasis, including multi-stage decompression of the common bile duct via an endoscopic retrograde cholangiopancreatography (ERCP) either before or after laparoscopic cholecystectomy (LC), or a single-stage LC with common bile duct exploration (CBDE). Single-stage LC with on-table ERCP (known as a rendezvous procedure) is another well-described but less common method. In Australia, multi-stage management still predominates despite recent studies showing single-stage procedures having comparable success rates with reduced length of stay (LoS) and higher levels of patient satisfaction [4]. A network meta-analysis by Ricci et al. [5] of 20 randomized-control trials found that the most common management plan was LC with CBDE (36.7%), followed by pre-operative ERCP with LC (35.3%) and rendezvous procedure (24.5%). They also reported the rendezvous procedure as the most likely to have the highest rates of success with the shortest hospital LoS; however, failed to demonstrate any superior approach when comparing morbidity outcomes between the groups.

Furthermore, the availability of specialist endoscopists is often limited in regional settings, echoing the benefit of single-stage procedures, which can be managed by general surgeons alone. Census data from 2021 reports that 28% of the Australian population lives in rural, regional, or remote locations, with only 15% of practicing surgeons living in these areas [6] and an even lower percentage of other medical specialties available. Therefore, it is important to rationalize the equitable use of resources and tailor management specific to the patient and the health service in which they are managed. Finally, ERCP carries significant risks, including pancreatitis [7], with post-ERCP rates of pancreatitis around 7%. Longer-term outcomes of ERCP, associated with the incidence of sphincterotomies, include reflux of duodenal contents, resulting in recurrent common bile duct (CBD) stones causing cholangitis [8], or increased incidence of biliary malignancy.

The aim of this study is to determine the primary management of choledocholithiasis in a regional setting, comparing patient management and outcomes of single-stage and multi-stage procedures. Secondary aims include identifying factors associated with the presence of confirmed choledocholithiasis and comparing the efficacy and morbidity of management options.

## Materials and methods

Study design

A single-center, retrospective, cross-sectional analysis was performed on all patients admitted to Toowoomba Hospital for management of diagnosed or suspected choledocholithiasis from January 2021 to March 2023. All procedures were conducted at Toowoomba Hospital, which offers ERCP within the Darling Downs Hospital and Health System, a network that encompasses over 290,000 people. A total of 495 consecutive ERCPs were performed during the inclusion dates, which were assessed for their indication and cross-referenced with clinical documentation. All patients who underwent an ERCP for suspected choledocholithiasis or who had a CBDE for choledocholithiasis were included. Patients were excluded if they met diagnostic criteria for primary choledocholithiasis (CBD stone with cholecystectomy and clearance over two years prior) or if they underwent an ERCP primarily to investigate or manage a malignancy.

Data collection

Patient demographics (including age, sex, admission date, comorbidities, BMI, and smoking status) and procedural data (serum blood tests, imaging modality, and findings, ERCP findings, operative interventions, and complications) were collated from The Viewer (Queensland Government Heath Provider Portal) by three investigators. The primary outcome of this study was the method of management in patients with choledocholithiasis (single-stage or multi-stage management). Secondary outcomes included the rates of incidental choledocholithiasis, imaging modality of diagnosis, serum biochemical predictors of choledocholithiasis, LoS, and number of admissions. Operative reports were carefully examined for the result of intra-operative cholangiograms to confirm choledocholithiasis, and images were reviewed by consultant surgeons if no documentation was found.

Reference ranges used were total bilirubin (<20 μmol/L), alkaline phosphatase (30-110 unit/L), gamma-glutamyl transferase (<55 unit/L), aspartate transaminase (<35 unit/L) and alanine transaminase (<45 unit/L). Pancreatitis was diagnosed in accordance with the revised Atlanta criteria [9], with at least two of the following documented: epigastric pain, radiological evidence of pancreatitis, or an elevated serum lipase above three times the upper limit of normal (<60 unit/L). Normal CBD diameter was defined as <8mm [10] for all ages.

Statistical analysis

Data was analyzed using Jamovi 2.3.21, with outcomes reported as mean and standard deviation, or median and interquartile range (IQR), and a log transformation of LFTs included to standardize skewed results. Subgroups were analyzed to assess for differences between single-stage and multi-stage management using a two-tailed Fisher's exact test, and a p-value of <0.05 was considered statistically significant.

Ethics

Local ethics approval was obtained via the Darling Downs Health Human Research Ethics Committee (EX/2023/QTDD/101325) in Queensland, Australia on 14 August 2023. All data was de-identified and stored on-site via a password-protected server.

## Results

**Figure 1:**
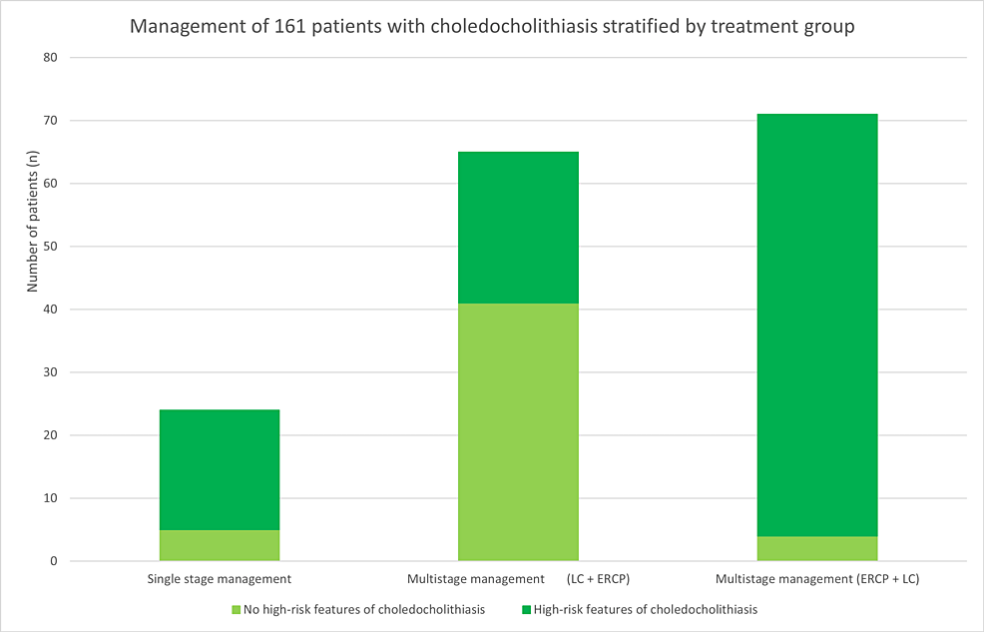
Number of patients managed per treatment group, stratified by the presence of high-risk features of choledocholithiasis on investigation LC - laparoscopic cholecystectomy; ERCP - endoscopic retrograde cholangiopancreatography

A total of 495 ERCPs were identified over 27 months, with procedural reports screened for indication and patients removed as per the exclusion criteria. Of the remaining ERCP procedures, 283 procedures (57.17%) were performed on 171 different patients. Multi-stage management was performed in 136 of the 171 patients (80.12%), with 34 patients (19.88%) undergoing ERCP alone and no surgical follow-up, typically due to poor surgical candidacy or patient preferences. A total of 195 patients were included across all groups, with 25 patients (12.82%) undergoing a single-stage procedure, including one combined LC and on-table ERCP. Patient summary characteristics are shown in Table 1 and were comparable between management groups. The number of patients undergoing each management type is shown in Figure 1.

**Table 1:** Summary characteristics of 195 patients admitted to a regional center with choledocholithiasis from January 2021 to March 2023 T2DM - type 2 diabetes mellitus; MI - myocardial infarction; COPD - chronic obstructive pulmonary disease; CKD - chronic kidney disease; CCF - congestive cardiac failure * Two patients had confirmed pregnancies during admission # History of one or more standard drinks per week

Characteristics	m	SD
Age (years), mean (SD)	56.4	21.3
Characteristics	n	%
Sex		
Male	77	39.5
Female*	118	60.5
Smoking status		
Current smoker	30	15.4
Ex-smoker	32	16.4
Non-Smoker	97	49.7
Not stated	36	18.5
Alcohol consumption		
Yes^#^	67	34.4
No	84	43.1
Not stated	44	22.6
Comorbidities		
T2DM	28	14.4
MI	16	8.2
COPD	15	22.6
CKD	15	7.7
CCF	7	7.7
Liver disease	9	4.6

The most common symptoms on presentation were abdominal pain (86.2%), nausea (45.6%), vomiting (34.9%), jaundice (15.4%) and fever (7.2%). Only 32 patients (16.4%) had a previous diagnosis of pancreatitis. The most common imaging investigation was a focused abdominal ultrasound scan (USS), which was ordered in 122 patients (62.6%), followed by an abdominal computed tomography scan (CT) in 45 patients (23.1%) and an abdominal magnetic resonance imaging scan (MRI) in 24 patients (12.3%). Initial investigations revealed choledocholithiasis in 81 patients (42.4%), with the majority of those only having a single stone (60.4%). Choledocholithiasis was not found on initial imaging in 110 patients (56.4%), with 31 patients (15.9%) having incidental choledocholithiasis diagnosed at the time of cholecystectomy with no pre-procedural imaging findings of biliary dilatation or a biochemically elevated bilirubin.

Biochemical markers were recorded on admission and pre-procedurally (including LFTs, bilirubin, and white cell count) and compared between patients with confirmed choledocholithiasis on index ERCP and those without. A logarithmic transformation was used, and serum markers were compared using an independent samples t-test, as seen in Table 2. There was strong evidence to suggest that the mean bilirubin of patients with confirmed choledocholithiasis on index ERCP is higher (36.5 μmol/L) than those without ERCP confirmed choledocholithiasis (19.7 μmol/L). Further non-parametric analysis via a Mann-Whitney U test revealed evidence of an association between the time to index ERCP and a positive finding of choledocholithiasis (U=1875, p=0.022), with patients who had a stone waiting an average of 4.98 days compared to 11.44 days in those who did not have stones noted.

**Table 2:** Independent samples t-test comparing the biochemical markers of 153 patients admitted to a regional center with choledocholithiasis, stratified by index ERCP findings ERCP - endoscopic retrograde cholangiopancreatography; AST - aspartate transaminase; ALT - alanine transaminase; GGT - gamma-glutamyl transferase; Bili - bilirubin Hₐ μ Negative ERCP ≠ μ Positive ERCP

	Statistic	df	p	Mean difference	SE difference	95% Confidence Interval	
						Lower	Upper
Log(Bili)	-2.7189	151	0.007	-0.2378	0.0874	-0.4105	-0.065
Log(AST)	-2.4992	151	0.014	-0.345	0.138	-0.6178	-0.0723
Log(ALT)	-2.2192	151	0.028	-0.1779	0.0802	-0.3364	-0.0195
Log(ALP)	-2.6382	151	0.009	-0.1609	0.061	-0.2814	-0.0404
Log(GGT)	-2.7498	151	0.007	-0.2348	0.0854	-0.4036	-0.0661

**Figure 2:**
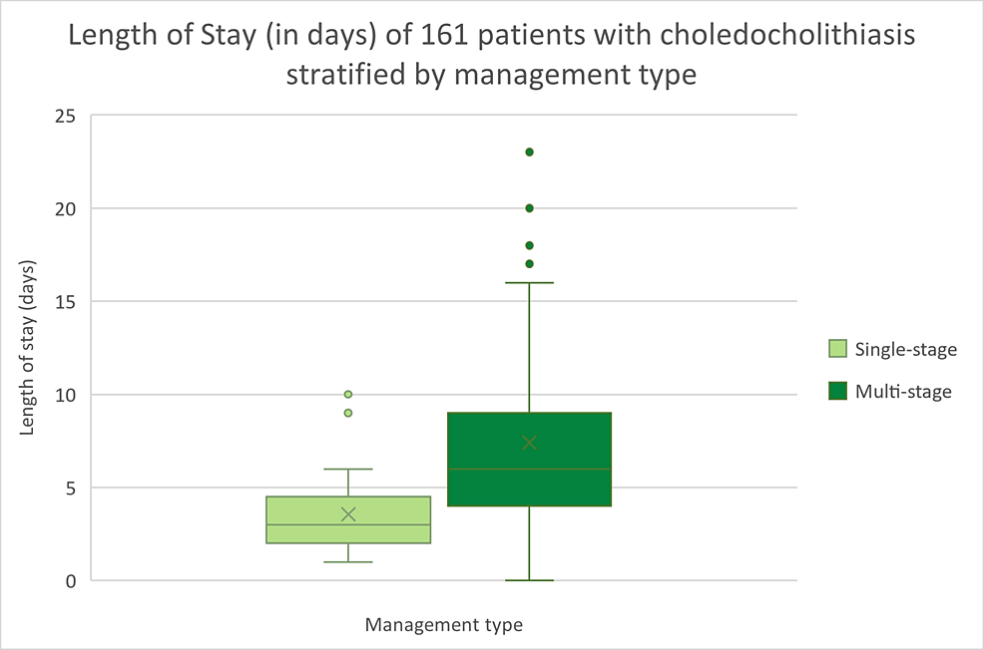
Length of stay (days) of patients with choledocholithiasis stratified by management type

Single-stage procedures had an 80% success rate with an average LoS of 3.6 days, compared to multi-stage procedures with an average LoS of 8.1 days (Figure 2) and an ERCP success rate of 93%. Of the patients who underwent a cholecystectomy, nine had a subtotal cholecystectomy, and three converted to an open procedure. An intraoperative cholangiogram was performed on 141 patients, with only 39 having a normal IOC. The most common abnormality noted was a filling defect in 73 patients (51.8%), with 26 patients (18.4%) noting no free flow into the duodenum, six patients (4.3%) having abnormal tapering of the distal CBD and four patients (2.8%) having abnormal proximal anatomy.

Complication rates, including post-ERCP pancreatitis, CBD injury, and hemorrhage, were comparable between groups, with ERCP having 11.7% and LC with CBDE having 9.7% complication rates. Post-ERCP complications included 19 episodes of ERCP-pancreatitis and a single episode of hemorrhage requiring endoscopic clipping. There were 20 recorded unsuccessful ERCPs, typically due to papillary edema or difficult anatomy with the presence of a duodenal diverticulum.

## Discussion

Predictors and pre-operative stratification of choledocholithiasis

In 2019, the American Society for Gastrointestinal Endoscopy (ASGE) published revised guidelines that attempted to risk stratify the likelihood of choledocholithiasis based on clinical predictors including age, LFT derangement, total bilirubin, presence of cholangitis, imaging reporting a dilated CBD or the presence of choledocholithiasis. However, subsequent validation studies have demonstrated only a modest predictive effect, with the presence of any high-risk features yielding a sensitivity of 65.8%, specificity of 86.3%, and accuracy of 70.4% [11]. A systematic review with the meta-analysis by Wang et al. [12] found a low weighted odds ratio for abnormal LFTs in predicting the presence of choledocholithiasis, as well as insufficient data to comment on the predictive nature of total bilirubin and CBD>6 mm, in accordance with the 2019 ASGE guidelines. A retrospective review of 3904 cholecystectomies by Ng et al. [9] did not find any routine pre-operative, radiological, or biochemical factors that reliably predicted the presence of choledocholithiasis defined by routine IOC. Contrarily, this study found strong evidence between total bilirubin alone and an initial positive ERCP, with cases of confirmed choledocholithiasis having higher total bilirubin and more likely to have stones on ERCP than those without (36.5 μmol/L vs 19.7 μmol/L).

Incidental choledocholithiasis and the natural history of stones

Incidental choledocholithiasis poses a significant and difficult management decision for surgeons, with incidences reported around 10% [2]. The ASGE guidelines also suggest that "pre- or post-operative ERCP or laparoscopic treatment be performed for patients at high risk of choledocholithiasis or positive IOC, depending on local surgical and endoscopic expertise". In our study, of the patients that had an upfront LC and were diagnosed at the time with incidental choledocholithiasis, five proceeded onto a CBDE with a 100% success rate while 26 underwent multi-stage management, with only 11 (42%) having a stone confirmed on ERCP. Furthermore, there is very little available evidence about the natural history of choledocholithiasis, with the ESGE guidelines published in 2019 "offering stone extraction to all patients with CBD stones, symptomatic or not, who are fit enough to tolerate intervention" [3]. The strong recommendations were based on low evidence, citing the GallRiks study of 3,969 patients with choledocholithiasis on IOC, 594 of which were left in situ, with 25.3% developing complications within four years compared to 12.7% in those with CBD stones removed [13]. The passage of smaller stones has been documented in several studies without complications, with Collins et al. reporting 24 of 46 patients having resolution despite choledocholithiasis confirmed on IOC without further intervention [14]. Our study found evidence of an association between the time to index ERCP and a positive finding of choledocholithiasis (U=1875, p=0.022), which may be contributed to in part by the spontaneous passage of choledocholithiasis. In the absence of stronger predictive tools that may select patients requiring intervention, single-stage procedures may prevent further unnecessary treatment, greater hospital costs, and increased patient length of stays compared to multi-stage procedures.

Multi-stage management of choledocholithiasis

There are multiple reported techniques in managing patients with choledocholithiasis, selected based on surgical and endoscopic availability and expertise, patient factors such as comorbidities, and reported safety and efficacy of each procedure. In this study, the majority of patients underwent a two-stage procedure, with the breakdown of management shown in Figure 2. In 2018, Ricci et al. [5] conducted a systematic review and network meta-analysis of 20 randomized clinical trials, including 2489 unique patients undergoing either LC and laparoscopic common bile duct exploration (LCBDE), LC, and post-operative ERCP, pre-operative ERCP, and LC or a rendezvous procedure. The study reported rendezvous procedures as having the highest efficacy, with single-stage management having the least associated costs and shorter operative time compared to multi-stage management. The clinical reasoning for a pre-operative ERCP in multi-stage management is based on concerns that the CBD stones will result in acute pancreatitis or a cystic duct leak secondary to raised intra-biliary pressure. A Swiss study [15] of 10,000 laparoscopic cholecystectomies found a post-operative acute pancreatitis rate of 0.34% and attributed only four cases to choledocholithiasis (0.0004%). The incidence of CBD stones in patients with cystic duct stump leaks is only 3-5%, and thus neither of these concerns is significant. Furthermore, as mentioned previously, the longer-term outcomes of ERCP sphincterotomies may be associated with recurrent CBD stones causing cholangitis or increased risk of biliary malignancy. Finally, multiple cost-analysis reviews have shown single-stage procedures to be clearly more cost-effective [16,17] in the management of choledocholithiasis and provide an incentive for a paradigm shift in modern management.

Management of choledocholithiasis in a regional setting

Management of choledocholithiasis in regional settings can often be challenging due to limited access to sub-specialist care, which may limit a health network's ability to provide quick access to specialized procedures. A recent review of 191 consecutive patients [18] who had an ERCP performed by a trained general surgeon in a regional center found that the procedure was completed well above published standards and was cost-effective when comparing overall management costs. Furthermore, despite perceived difficulties surrounding a CBDE, Hodgson et al. [19] reported that a caseload of 70 CBDE over 10 years was sufficient in producing an 80% success rate, with only one procedure a year needed to maintain a safe procedure with a 50% success rate.

## Conclusions

The management of choledocholithiasis varies and depends on patient, clinician and hospital factors. Incidental choledocholithiasis presents a significant burden of disease and likely benefits from early management. Single-stage management is likely superior in reducing patient length of stay, overall admission costs and improved patient satisfaction when compared to multi-stage management. General surgeons in regional settings should be supported to expand their skills to safely provide safe and effective services to patients in regional areas.
